# Supplementary figures and images for: Looking for Crumbs in the Obesity Forest: Anti-obesity Interventions and Obesity-Associated Cardiometabolic Traits in the Mexican Population. History and Systematic Review With Meta-Analyses
Source: Front Med (Lausanne). 2021 Nov 3;8:665023. doi: 10.3389/fmed.2021.665023 (PMC8595206; doi:10.3389/fmed.2021.665023)

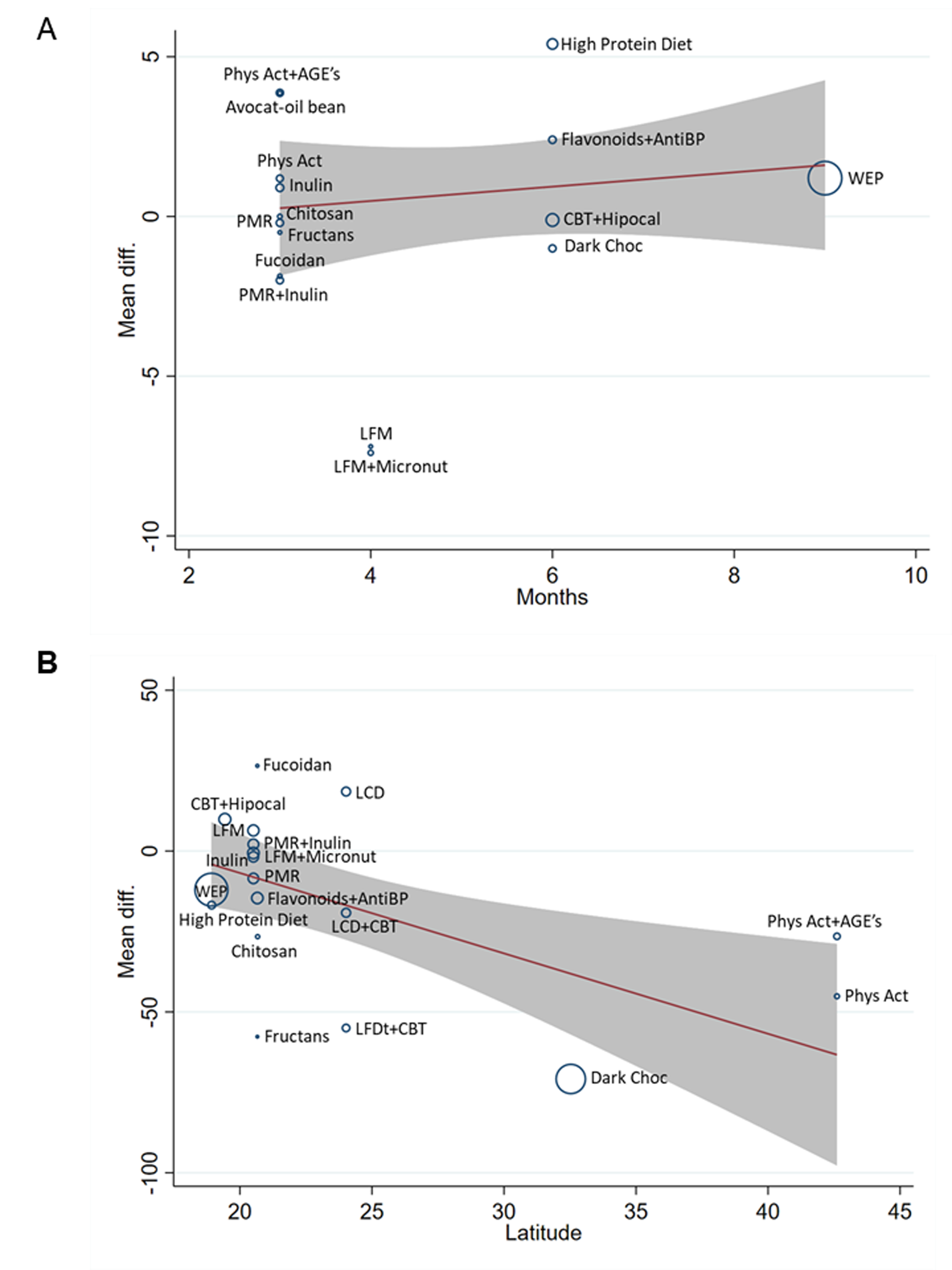

Supplement: Supplementary Figure 1 — Meta- regression of nutritional/behavioral effects on the mean difference in HDL-C (A) and triglycerides (B) concentrations, adjusted by mean age, BMI, duration of treatment (months), geographical latitude, and use of placebo or active comparator. The gray zone represents the 95% CI of the regression. Liraglutide was used in participants with the highest obesity and in geographic locations in northern Mexico. AGE's, Advanced glycation end-product; AntiBP, Antiblood pressure medication; CBT, Cognitive-behavioral therapy; Dark Choc, Dark chocolate, Hipol; LCD, Low carbohydrate diet; LFDt, Low fat diet; LFM, Low fat milk, Micronut; Phys Act, Physical activity; PMR, Partial meal replacement; WEP, Water and education provision. [file Image_1.TIF]

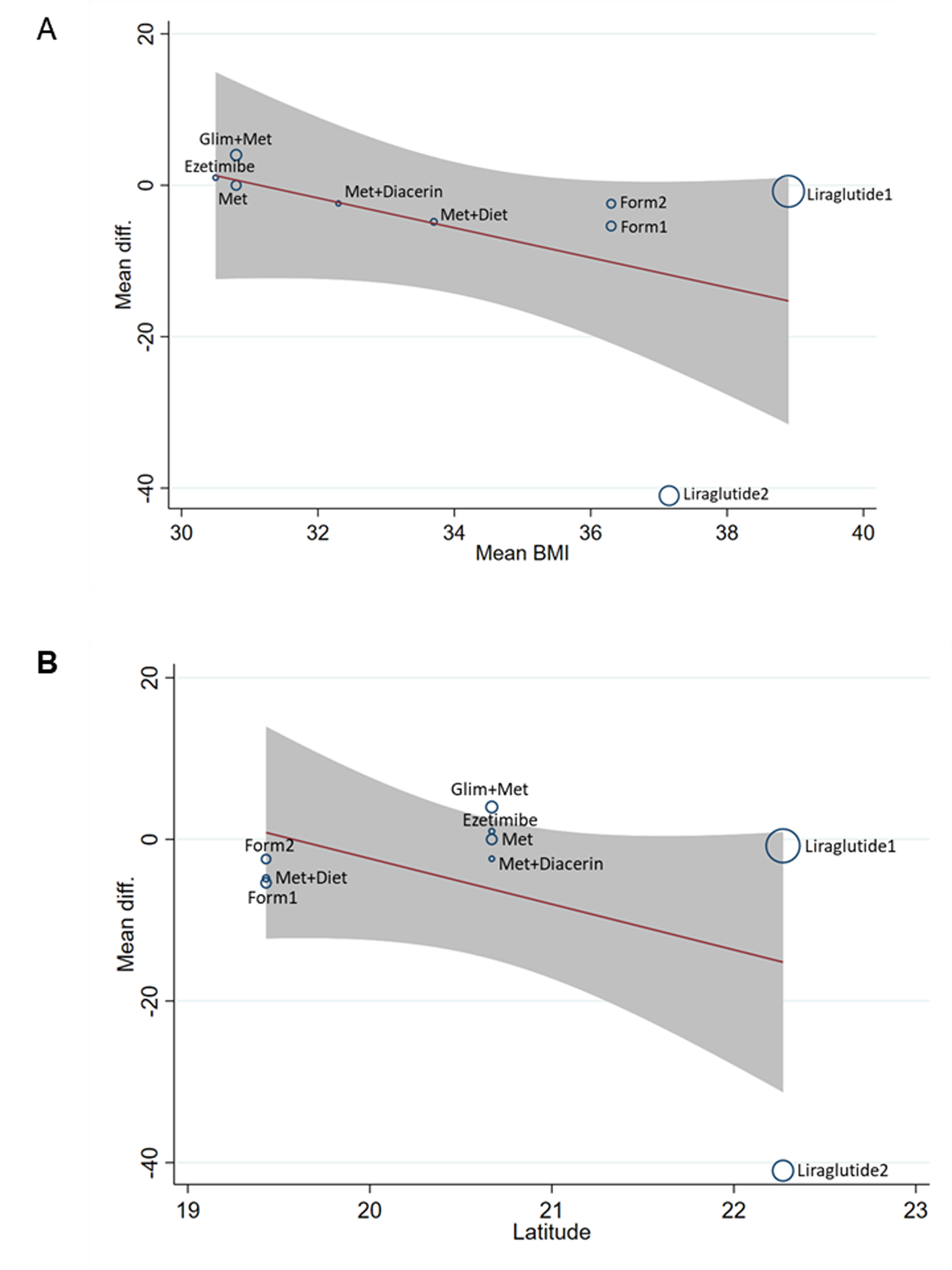

Supplement: Supplementary Figure 2 — Meta regression of medication effects on the mean difference in diastolic blood pressure adjusted by mean age, BMI, duration of treatment (months), geographical latitude, and use of placebo or active comparator. The (A) shows the effect of BMI and the (B) the geographical location. The gray zone represents the 95%CI of the regression. Liraglutide was used in participants with the highest obesity and in geographical locations in northern Mexico. The Form1 and Form2 are described in the text, they are not approved by FDA. Met, Metformin; Sibut, Sibutramine; Orlit, Orlistat; Glim, Glimepiride; Phent, Phentermine; Top, Topiramate. [file Image_2.TIF]

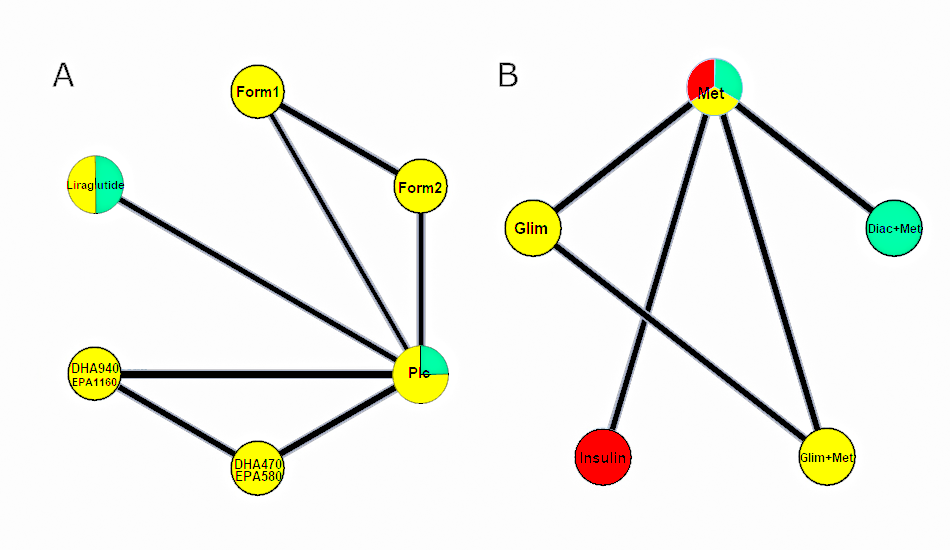

Supplement: Supplementary Figure 3 — Network meta-analysis of studies examining the efficacy of drug treatments in patients with obesity on (A) BMI in non-diabetic patients compared to placebo, (B) BMI in patients with diabetes compared to metformin. The colors of the edges and nodes refer to the risk of bias: low (green), moderate (yellow), and high (red). DHA and EPA doses are in mg per day. Met, Metformin; Diac+Met, Diacerin + Metformin. The Form1 and Form2 are described in the text, they are not approved by FDA but approved by its Mexican counterpart, COFEPRIS. Plc, Placebo. [file Image_3.TIF]
